# Genetic Correction of Stem Cells in the Treatment of Inherited Diseases and Focus on Xeroderma Pigmentosum

**DOI:** 10.3390/ijms141020019

**Published:** 2013-10-09

**Authors:** Sophie Rouanet, Emilie Warrick, Yannick Gache, Sabine Scarzello, Marie-Françoise Avril, Françoise Bernerd, Thierry Magnaldo

**Affiliations:** 1 Genetics and Physiopathology of Epithelial Cancers, INSERM U 1081-CNRS UMR 7284-UNS, Institute for Research on Cancer and Aging, Nice, Medical school, 28 Avenue de Valombrose, 06107 Nice Cedex 2, France; E-Mails: srouanet@unice.fr (S.R.); gache@unice.fr (Y.G.); scarzell@unice.fr (S.S.); 2 L’Oréal Research and Innovation, 92217 Clichy, France; E-Mails: ewarrick@rd.loreal.com (E.W.); fbernerd@rd.loreal.com (F.B.); 3 Hopital Cochin, Pavillon Tarnier, APHP, Université Paris 6, 75006 Paris, France; E-Mail: marie-francoise.avril@cch.aphp.fr

**Keywords:** stem cells, skin, epidermis, cancer, DNA repair, genetic correction, xeroderma pigmentosum

## Abstract

Somatic stem cells ensure tissue renewal along life and healing of injuries. Their safe isolation, genetic manipulation *ex vivo* and reinfusion in patients suffering from life threatening immune deficiencies (for example, severe combined immunodeficiency (SCID)) have demonstrated the efficacy of *ex vivo* gene therapy. Similarly, adult epidermal stem cells have the capacity to renew epidermis, the fully differentiated, protective envelope of our body. Stable skin replacement of severely burned patients have proven life saving. Xeroderma pigmentosum (XP) is a devastating disease due to severe defects in the repair of mutagenic DNA lesions introduced upon exposure to solar radiations. Most patients die from the consequences of budding hundreds of skin cancers in the absence of photoprotection. We have developed a safe procedure of genetic correction of epidermal stem cells isolated from XP patients. Preclinical and safety assessments indicate successful correction of XP epidermal stem cells in the long term and their capacity to regenerate a normal skin with full capacities of DNA repair.

## 1. Stem Cells

From the early embryo to the adult, development, renewal and repair of tissues or organs depends on the presence of stem cells, their proper recruitment and commitment towards differentiation. In 1979, Lajtha proposed that a stem cell “has the capability to renew for the duration of life, a fully functional tissue or organ from which it originates” [1]. Over the last decades, however, experimental evidences have shown that stem cells fall in distinct categories as a function of their potential to found one or several types of cell lineages that is to be toti-, pluri-, oligo- or uni-potent.

Totipotent stem cells have the capability of generating any somatic cell lineage of the body including embryonic membranes. Embryonic stem cells (ESC), derived from the inner mass of the blastocyst, are totipotent *in vivo. In vitro*, ESC cells may be pushed towards specific ways of differentiation such as cardiac, neural or blood cell, insofar appropriate feeder cells and specific culture conditions are set up [2]. Somatic stem cells may be pluri- to uni-potent but remain unable to generate embryonic membranes. They are thought to reside within topological “niches” composed of specific biochemical components including extra cellular matrix macromolecules, growth factors and stromal cells. Hematopoietic stem cells (HSC) have probably been best characterized among stem cells. These cells are multi-potent and give rise to lymphoid and myeloid precursor lineages [3]. Other stem cells, such as those located in the hair follicle (HF) may also give rise to several precursor cells. Barrandon and colleagues showed that HF stem cells are located in a delimited region (the bulge) and give rise to interfollicular (IF) epidermis, HF and sebaceous units [4,5]. Under these experimental circumstances, these epidermal stem cells are thus tri-potent. Finally, stem cells may also be programmed to lead to a single type of progeny. This is the case in epidermis where adult epidermal stem cells located in the basal layer of IF areas exhibit unlimited life span but give rise to a progeny whose ontogenic capacity is limited to glabrous epidermis without any of the skin appendages. These stem cells are thus said to be uni-potent.

## 2. Stem Cells and the Microenvironment

The fate of stem cells does not solely depend on their intrinsic characteristics but also on the environment they interact with. Since the historical experiment of Spemann published in 1924, we know that this may hold true from early development to the adult life [6]. According to a strategy of interspecific grafts, there emerged the concept that the developmental fate of a group of cells may be drastically changed by the interaction with a recipient substrate.

During the integument development, mesodermal cells contribute to the formation of the dermis while ectodermal cells form the epidermis as well as epidermal appendages. The dermis constitutes a physical support and a source of nutriments for epidermis. The epidermis is a stratified squamous epithelium in contact with the external environment and connected to the dermis via a complex basement membrane. Upon development, the dermis and the epidermis evolve in a highly coordinated manner*,* leading to the basic structure of an organ endowed with functional properties adapted to local body requirements such as the presence of HF, highly “flexible” areas covering joints (papillomatosis over knee, elbow), or thick cornification in the palmoplantar skin. Further, to assess the role of fibroblasts in HF development, Reynolds and Jahoda reported that transplantation of fibroblasts isolated from the rat dermal papilla of HF may confer to IF keratinocytes the capacity to form hairs [7]. The type of ectodermal differentiation is also dependent on the mesodermis. Pearton and colleagues showed that association of corneal epithelium with a hair-forming dermis results in condensation of dermal cells, and subsequent development of a hairy epidermal structure expressing characteristic follicular markers [8]. Recent work from the group of Yann Barrandon showed that epithelial cells found in the Hassal corpuscule of the mouse thymus can regenerate epidermis, hair follicles, and sebaceous glands when transplanted onto the dermal skin compartment [9]. These experiments illustrate again the inductive role of the microenvironment on genetic and epigenetic programs controlling the fate of stem cells. The plasticity of ectodermal cells also depends on their stage of development. For instance, grafting of epithelial sheets of adult human keratinocytes isolated from palmoplantar skin onto non-palmoplantar body area (foreleg) results in the development of an epidermis retaining expression of the palmoplantar specific marker keratin K9 [10].

In summary, stem cells may derive from either embryonic or adult tissues, and their fate and potency may be influenced by microenvironmental factors. Based on their regeneration capacity, stem cells have long been thought as valuable tools to compensate the loss of an essential biological function in degenerative and/or genetic diseases. Here, we focus on somatic stem cells and the possibility to use them as transplantable recipients of therapeutic genes. Special emphasis is made on epidermal stem cells and perspectives of treatment for monogenic genodermatoses, notably xeroderma pigmentosum.

## 3. Epidermal Stem Cells

In 1975, Rheinwald and Green reported that epidermal keratinocytes, isolated from a human healthy biopsy, could be cultured in the long term, provided the presence of murine embryonic γ/X-ray lethally irradiated 3T3 fibroblasts used as a cell feeder layer [11]. The irradiated cells constitute a surrogate of the niche: they no longer proliferate, secrete growth factors, cytokines and extracellular matrix components that are essential for the growth of a population of primary keratinocytes containing stem cells. Among growth factors necessary for the growth of keratinocytes is epidermal growth factor (EGF) [12]. EGF is essential for the propagation of keratinocytes subpopulations of over 100 population doublings (PD) which represent a progeny of about a 10^21^ cells. Clonal analyses by Barrandon and Green led to the identification of three types of colonies according to morphological and growth characteristics: holoclones, meroclones and paraclones [13]. Holoclones correspond to colonies with a smooth perimeter of a size from 4 to 5 mm in diameter after 12–14 days of culture. Most of the cells (>95%) composing the holoclone colonies are also clonogenic and will generate a clonogenic progeny. On the opposite, paraclones are small sized abortive colonies (<1 mm) initiated by a cell with a growth potential limited to 15 divisions. Less than 5% of the descendent cells of a paraclone are themselves clonogenic: their lifespan is limited to about five divisions. Finally, meroclones form colonies of intermediate size (1 < *d* < 4 mm) with an irregular shape and a progeny comprising between 5% and 95% of clonogenic cells.

So far, clonal analysis remains a valuable and faithful tool to detect and estimate the proportion of stem cells in epidermis or in a population of cultured keratinocyte. On these bases, Gallico, Green, and colleagues were the first to show that under appropriate culture conditions, epidermal stem cells present in a 2 cm^2^ skin biopsy allow the development of epithelial sheets suitable for full surface skin replacement in severely burned patients [14].

## 4. Stem Cells in the Organization of the Epidermis

About 10% of cells located in the basal epidermal layer are cycling and can be localized by metabolic incorporation of DNA precursors or after antigenic labelling of cycling cells (e.g., Ki67, BrdU, H3TdR). For this reason, the basal layer is called *stratum germinativum*. However, since stem cells are rarely dividing *in vivo*, the ratio of stem to proliferative basal cells is low and only 0.1%–1% of basal cells are considered as *bona fide* stem cells. As early as in 1974, pioneer studies by Chris Potten and colleagues reported that after injury, the progeny of a recruited stem cell is organized as an epidermal proliferation unit (EPU) composed of transient amplifying (TA) and terminally differentiated cells [15–17]. According to these observations, it is believed that recruitment of a single stem cell may give rise to a cell compartment of transient amplification. Taïchman and colleagues, using xenograft of human epidermal keratinocytes, showed that human epidermal stem cells are dispersed along the basal compartment and demonstrated the presence of EPU in human skin [18,19]. More recently, however, Clayton and colleagues suggested that a compartment of TA cells is not essential to replenish mouse tail epidermis. According to this study, a stem cell could also directly give rise by asymmetric division to both a daughter stem cell and a cell triggered to terminal differentiation. Whether this model established from mouse tail skin (an alternation of ortho and parakeratotic segments of epidermis) can be generalized to epidermis remains unknown [20].

## 5. Follicular and Interfollicular Stem Cells

The HF resembles a full organ entity with a cycling life schematically made of growth, involution and rest phases called anagen, catagen, and telogen, respectively. The anagen phase depends on the presence of stem cells that travel all the way down to the hair matrix where they proliferate and fuel growth of the hair shaft. The life and “apparent death” cycle of HF early raised the question of the topologic location of HF stem cells. Most contribution in the field ensued from studies of hair pelage and vibrissal follicles in rodents. Based on the belief that stem cells are rarely dividing in their niche, mice were injected with DNA precursors such as BrdU or H3TdR before a two months cold chase. Under these circumstances, cells that retained the DNA label were localized in the bulge area of the outer root sheet as well as sparsed in the IF epidermis [21]. Later on, microdissection experiments showed that 95% of clonogenic cells of the hair are clustered in the bulge area of the adult rat vibrissal follicle. The other 5% clonogenic cells mostly derive from the bulb of the HF which contains rapidly proliferating cells from the hair matrix. *In vivo*, labeled rat bulge cells transplanted onto the immunodeficient mouse generated HF epidermis, sebaceous gland and hair, indicating the multi-potency of HF stem cells [22]. Interestingly, multiple labeling with distinguishable DNA precursors showed that HF stem cells contribute to the regeneration of IF epidermis and epithelial compartment of the pilosebaceous unit in injured hairy skin [22–24]. Thus, HF stem cells participate to interfollicular regeneration after injury but cannot be considered as *bona fide* epidermal stem cells at the steady state. Together, these seminal experiments showed that a large knowledge on epidermal stem cells ensued from studies of murine HF stem cells.

## 6. How to Fish Them Out: Markers of Epidermal Stem Cells?

To recognize and isolate epidermal stem cells is an issue in anticancer and gene therapy research and applications. Indeed, most skin cancers are derived from these cells that should be targeted by both pharmacological and gene-based therapies. Most studies have aimed at characterizing HF stem cells isolated from areas known to shelter them (*i.e.*, the HF bulge) and some markers have been identified. The K15 keratin was reported to be specifically over-expressed (×2) in the bulge and in basal cells located in the rete ridges of the IF epidermis [25,26]. As well, genome expression studies in cells isolated from the human HF showed slight CD200 mRNA increase [27,28]. Based on the notion of niche, other studies on IF keratinocytes have proposed that stem cells may exhibit specific adhesion properties. In 1982, Sun and Lavker already noticed that “serrated keratinocytes” were clustered in specific areas of the skin, such as deep rete ridges in palmo plantar epidermis and in epidermis at the tip of dermal papillae in breast skin. Serrated keratinocytes were supposed to be more strongly anchored to the basement membrane [29]. Since then, Adams and Watt showed that human epidermal cells isolated from fresh human samples adhere more rapidly to certain extracellular matrix components, notably collagen IV [30]. These cells also expressed higher levels (about ×2) of cell adhesion proteins such as β1 or α6 integrins [31–33] suggesting that strong anchoring to the basement membrane might be characteristic of stem cells. However, the levels of expression of these cell surface proteins are almost uniformly high under culture conditions and can poorly contribute to the discrimination of clonogenic cells. Since stem cells divide infrequently, criteria for stem cell purification were thus refined by considering their low levels of proliferation and the expression of growth factor receptors such as EGFR and transferrin receptor (CD71) [34,35]. Using multiple labeling of human skin and a sophisticated mathematical model, Martin and colleagues concluded that epidermal stem cells would share the pattern: high expression of p63, CD29+, CD49f+, MCSP, β-catenin, CD71 and Ki67, and would account for about 6% of basal cells [36]. Table 1 combines cell surface candidate markers of stemness and illustrates the fact that neither a single marker nor a truly consensual set of markers could support specific purification of stem cells.

## 7. Stem Cells in *Ex Vivo* Gene Therapy

*Ex vivo* gene therapy aims at correcting a biological defect for which there is no efficient therapeutic treatment. Sustainability of the cure not only depends on vectors used for gene transfer, but also on the cells destined to genetic manipulation. There are schematically three theoretical possibilities for obtaining therapeutic cells: embryonic stem cells (ESC), induced pluripotent stem (IPS) cells, and somatic stem cells.

ESC has long been thought promising to generate specific cell lineage. For instance, the possibility to trigger ESC towards neuronal differentiation has prompted perspectives of regeneration of specific brain areas suffering from neuro degenerative affections such as Parkinson or Alzheimer diseases [43–46]. ESC, however, may form teratoma after implantation in the laboratory mouse [47], which constitute a limitation in using them as therapeutic tools. Several studies suggested that growth and differentiation of teratomas are graft-site dependent [48,49]. Modeling components of extra cellular matrix would perhaps modify the fate of ESC towards determined cells lineages, and hence, support clinical applications.

More recently, successes in “resetting” somatic cells back to pluripotent stemness have raised much hope toward genetic manipulation of stem cells. IPS cells may be obtained from somatic cells by forcing the expression of four key factors identified in ESC: KLF4, cMyc, Oct3/4, and Sox2 [50]. Human adult skin keratinocytes and fibroblasts were among the first cells reprogrammed as pluripotent stem cells [51,52]. Living chimeras could be obtained from IPS cells [53]. As ESC, IPS cells may also be recipient of genetic manipulations aiming at gene correction, extinction or mutagenesis. In spite of potential safety issues *in vivo*, IPS cells thus represent tremendous potential for disease modeling, mechanistic deciphering, and innovative pharmacological approaches.

To date, somatic stem cells remain the favorite targets to further explore routes of genetic correction in human diseases. Most notably, hematopoietic and epidermal stem cells have been extensively studied and probably best illustrate the successes of *ex vivo* genetic correction.

## 8. Candidate Diseases for *Ex Vivo* Genetic Correction

**Figure 1:**
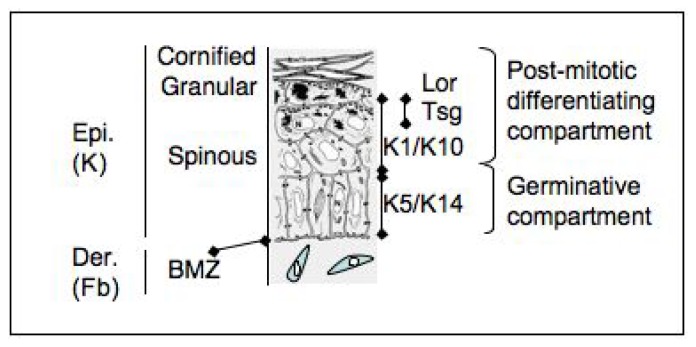
Schematic representation of human epidermis. Cell layers and corresponding abbreviations are indicated. Note that the basal epidermal layer, also called *stratum germinativum*, contains stem cells endowed with potential of full thickness epidermal regeneration. Conversely, the suprabasal compartment does not normally shelter proliferative cells but is characterized by a sequential program of genetic expression as attested by stepwise expression of markers, as indicated. BMZ, basement membrane zone; Epi, epidermis; Der, dermis; K, keratinocytes; Fb, fibroblasts; Kx, keratin X; Lor, Loricrin; Tsg, membrane bound transglutaminase.

Schematically, three types of disease are thought appropriate candidates for corrective gene transfer using epidermal keratinocytes: (1) systemic diseases such as hemorrhagic deficiencies [54], insulino-dependent diabete [55] and leptin-dependent obesity [56]. In these cases, although skin or epidermis do not necessarily express overt phenotypic traits of the disease, modified epidermal stem cells may serve as a reservoir for stable delivery of diffusible substances in the blood stream; (2) diseases with main expression of traits in skin (genodermatoses) resulting from defects in proteins such as keratins, loricrin, epidermal transglutaminase, cell envelope precursors, integrins, collagens, or protease inhibitors; (3) diseases due to alteration of ubiquitously expressed genes with special impact in skin. These include gene products involved in essential processes of the control of cell growth (P63, ectodermal dysplasia), transcriptional regulation (NEMO/IKKγ, *incontinenta pigmenti*; PATCHED, nevoid basal cell carcinoma or Goltz-Gorlin’s syndrome), telomeric (congenital dyskeratosis), and non-telomeric genome maintenance (xeroderma pigmentosum). Figure 1 is a schematic representation of a section of human skin. Table 2 combines representative examples of those diseases.

Even though non-exhaustive, these illustrations underline the complexity of genetic intervention in the case of diseases exhibiting both skin alterations and developmental and/or neurodegenerative traits. For instance, patients suffering from the XP-D form of XP suffer both from photosensitivity and neurologic afflictions. In contrast, genetic diseases with symptomatic expression in skin due to disabled production of structural proteins may appear, *a priori*, as better candidates toward gene-based therapeutic approaches.

## 9. Specifications

### 9.1. Inheritance and Mutation Typology

The feasibility of gene-based curing of a specific cell defect depends on its mode of inheritance. In the case of dominantly inherited diseases, haploinsufficiency (e.g., Gorlin syndrome) may theoretically be counterbalanced by a simple complementation procedure allowing restoration of proper levels of the limiting protein. This implies that after complementation, the mutated allele is not expressed at a high enough level to perturb proper signaling of a given pathway. In this respect, nonsense mutations that introduce premature stop codons are rapidly eliminated by nonsense mediated mRNA decay; reexpression of the WT allele may then be sufficient for complementation. Conversely, missense mutations give rise to erroneous proteins that expression should be replaced by their WT form. Compensating biallelic nonsense mutations responsible for recessively inherited genetic diseases still remains much easier than eliminating expression of a false sense mutation.

### 9.2. Safety

In the last decade, safety issues have been of increasing importance in the field of corrective gene transfer. In the 1980s, Howard Green was called by Gregory Gallico, a plastic surgeon, to perform grafting of cultured epithelial sheets to two young brothers suffering from 95% severely burned skin [14]. At this time, there was neither authorization nor staff dedicated to such a surgical intervention. In front of such a matter of conscience, it was agreed that there was also no right to let the two boys die. Thanks to the mobilization of Dr. Green’s staff, the children were grafted. Today, the “Green’s method” is being used routinely and has saved thousands of burned people worldwide.

In 2000, Marina Cavazzano-Calvo, Alain Fisher, and their colleagues reported the first genetic correction of infants (<5 years) suffering from lethal severe combined immunodeficiency (SCID-X1) [57]. This success relied on the purification of hematopoietic stem cells (HSC) using CD34 labeling which allows purification of progenitor cells, transduction of CD34+ HSC with high titer MFG γ retroviruses expressing the therapeutic gene and finally, patient infusion to reconstitute normal hematopoietic lineages. Worldwide, 28 of 31 children were cured [57–60] while five developed leukemia due to inappropriate cis-activation of the *LMO-2* proto-oncogene and clonal T-cell proliferation [61]. One patient died but others were cured by chemotherapy and retained benefits of the genetic intervention. Even though the approach was highly successful, clinical trials relying on gene transfer based on relatively basic retro or lentivirus backbone were interrupted.

To avoid cis-activation of inappropriate genes, new generations of vectors, either derived from γ-retrovirus or lentivirus, were developed. In brief, these vectors are defective for the replication (by itself, the infected cell cannot produce infectious viral particles), bear a 3′ LTR deleted from the U3 sequence, a WPRE sequence (woodchuck post-transcriptional regulatory element), and an internal promoter chosen for its short size and appropriate strength. This configuration favors transcription from the internal promoter while the enhancer activity of the 5′ LTR is strongly attenuated to avoid distal *cis*-activation (potentially of proto-oncogenes). Using such an improved lentivirus generation, Cartier and colleagues reported significant clinical benefits in two patients suffering from Adrenoleukodystrophy (ALD, a severe demyelinating disease) [62]. As for SCID infants [57], CD34+ HSC were purified from ALD patients prior to lentiviral transduction and reinfusion. Clinical benefits were observed 14–16 months later with the discontinuing of progressive demyelinisation and polyclonal reconstitution of haematopoietic lineage expressing the ALD therapeutic protein. Thanks to specific improvement of the therapeutic protocols (including notably, patient reduced intensity myeloablative regiment using busulfan and fludarabine) Aiuti and colleagues recently reported successful curing of patients suffering from the Wiskott-Aldrich syndrome (WAS). Proper expression of the therapeutic *WAS* gene in hematopoietic stem cells was made possible using an optimized lentiviral vector harboring the endogenous *WAS* promoter [63].

### 9.3. Selection

The need for selection of successfully transduced cells (*i.e.*, showing normalization of the functional defect) depends on whether or not the fraction of corrected cells is sufficient to complement the phenotype, for instance, in the case of a secreted protein. Alternatively, recovery of expression of a therapeutic gene in a fraction of stem cells may also confer growth advantage and clonal expansion of corrected populations. The need for selection of transduced cells also depends on whether or not the genetic defect may be accompanied by cancer proneness. In the latter case, non-corrected cells should be carefully eliminated before grafting. Altogether, these examples indicate how approaches of genetic correction should be flexible enough to fit disease specifications.

## 10. Corrective Gene Transfer in Primary Epidermal Keratinocytes from Patients Suffering from Xeroderma Pigmentosum

Over the last decades, the possibility to produce genetically manipulated keratinocytes and regenerate pieces of repaired epidermis *in vitro* have motivated huge amounts of work toward *ex vivo* cutaneous genetic correction. The main challenges in the field have focused on: (1) the elaboration of vectors fulfilling the safety concerns inherent to manipulation of the genome of stem cells; (2) obtaining pure corrected stem cell populations; (3) the improvement of graft procedures.

Skin is probably one of the most stressed organs. It is exposed to mechanical constraints inherent to its cover function but it must exhibit high flexibility. Skin is also exposed to external microorganisms, temperature variations, and radiations. Ultraviolets from the sun can be considered as the prime insult to skin. UVA and UVB may travel across skin layers where their energy is absorbed by chemical and biochemical components. In the DNA, UVB, and to a lesser extent UVA, introduce lesions at dipyrimidine sequences (CC, TT, CT, and TC) located in the same DNA strand. Some lesions (the Cyclobutane pyrimidine dimers (CPD)) distort the DNA, block transcription and replication processes, and trigger the DNA damage response. Other lesions (the 6-4 pyrimidine pyrimidone photo products (6-4 PP)) are specifically induced by UVB and account for about 20% of dipyrimidine sequences. Both CPD and 6-4 PP are mutagenic upon replication, leading to C to T transitions and CC to TT tandem mutations. These mutations constitute a large part of UV mutagenesis found in sun exposed skin tumors.

### 10.1. Nucleotide Excision Repair of DNA and Related Diseases

From bacteria to humans, species have developed protection systems dedicated to repair UV induced DNA lesions. Nucleotide excision repair (NER) is a versatile mechanism whose activity is centered on removal of bulky DNA adducts. NER thus not only plays a role in the skin but also in other organs such as lungs that are exposed to mutagenic substances contained in air or smoke. NER proceeds through sequential steps, starting with: (1) the recognition of the distortion by XPC, DDB2, and XPA; (2) a multiprotein complex assembles and is stabilized around the lesion. This complex is centered on TFIIH (basal transcription factor II H) and contains the two ATP dependent DNA helicases XPB and XPD; (3) XPB and XPD unwind the DNA around the lesion; (4) the XPG and XPF/ERCC1 DNA endonucleases incise DNA strand containing the lesion; (5) the DNA gap (protected by single strand DNA binding proteins) is filled by 5′–3′ replicative synthesis by the ɛ/δ polymerases; (6) the neosynthesized strand is 3′ ligated. When the lesion is located on the transcribed strand of an active gene, the step of recognition is signaled by stalling of the RNA polymerase II. In this case, all cell transcription is stopped, suggesting a mechanism of amplification.

NER is a faithful mechanism, leaving the genome intact, and hence, is essential for fighting tumorigenesis. To the contrary, dysfunction of NER due to germinal mutation in one of its pivotal genes is responsible for the xeroderma pigmentosum disease and the other developmental/ photosensitivity syndromes trichothiodystrophy and Cockayne syndromes [64–67]. The XP disease falls into seven groups of genetic complementation (XP-A to XP-G) due to mutations in *XPA* to *XPG* genes. *XPC* mutations make up about 50% of XP patients. All XP patients present with severe sun sensitivity, skin freckles, and early onset (by 8 years) of skin tumors in sun exposed areas. For not yet understood reasons, tumors not only develop at an early age, but also are more aggressive than in the general population. Namely, the ratio of spinous cell carcinomas (SCC) and malignant melanomas (MM) (that both give rise to metastasis) to basal cell carcinomas (BCC) is about 5/1 in XP patients while it is rather 1/5 in the general population.

Clinical care of XP patients includes strict sun avoidance, skin protection, and frequent surgical resection of suspect lesions such as actinic keratoses. Skin dilapidation may frequently require plastic reconstructive surgery [68] by autologous grafting of uninvolved skin which, nevertheless, remains prone to UV-induced carcinogenesis.

### 10.2. Genetic Correction of Xeroderma Pigmentosum

Alike other genodermatoses, such as epidermolysis bullosa, lamellar ichtyosis, or Netherton syndrome, the absence of treatment of the XP has motivated extensive researches towards genetic correction of skin cells, most notably of keratinocyte from which epidermal cancers develop. Pioneer studies principally addressed this question using dermal XP fibroblasts (XP-A, XP-C, XP-D) that are less demanding with respect to cell culture requirements [69–71]. We thus set up cell culture conditions for XP keratinocytes. We paid special attention to the XP-C group of genetic complementation. First, XP-C patients account for about 50% of XP cases in our Earth region (Europe and North Africa). Second, clinical traits of XP-C patients are mostly limited to skin and eye manifestations. Finally, XP-C patients are, in principle, exempt from neurological disorders described in other groups. Of note, a recent report by Hadj-Rabia and colleagues suggested that absence of the XPC protein may result in systemic manifestations including internal cancers with yet uncharacterized etiology [72]. In accordance, organotypic skin cultures comprising an XP-C dermal equivalent over-express the matrix metalloproteinase MMP1 and provoke epidermal cell invasions [73]. Thus, although virtually all skin cancers in XP patients derive from keratinocytes and melanocytes, the role of stromal fibroblasts must also be considered. Benefits of a genetically corrected epidermal compartment could be further improved by complement pharmacology targeting dermal fibroblasts.

### 10.3. Mandatory Selection: Exploiting Differentiation/Proliferation Features of Human Epidermal Keratinocytes

Obtaining pure populations of genetically corrected cells before grafting is mandatory provided the susceptibility of XP-C cells to carcinogenesis. The use of antibiotic resistance genes, most of which are derived from microorganisms, cannot be considered and, in any case, introduction of foreign selection molecule constitutes substantial risk of immune reaction against selected cells. For these reasons, population of genetically corrected cells re-infused [57] or grafted in human patients [10] or in immunocompetent animals [74] were not selected. Selection of corrected XP keratinocytes using the TN5 bacterial gene conferring geneticin resistance was accompanied with loss of transgene expression after six to eight weeks of culture [75].

**Figure 2:**
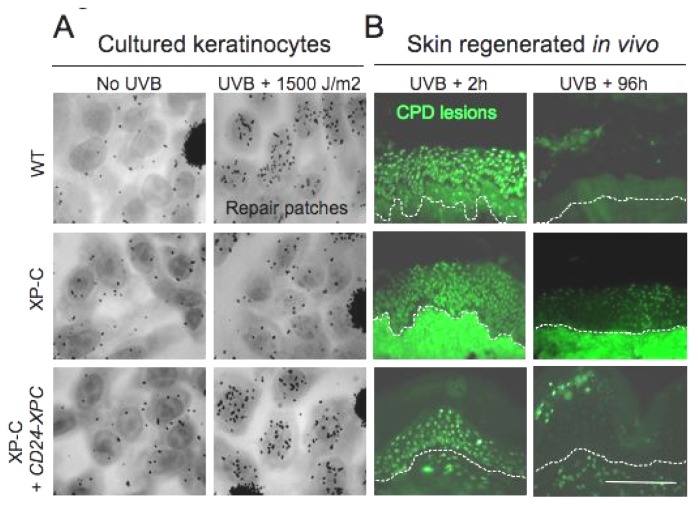
Genetically corrected XP-C keratinocytes exhibit full DNA repair capacity *ex vivo* and *in vivo* in regenerated skin. (**A**) Primary epidermal keratinocytes were isolated from control (WT) or XP-C (XP-C) individuals. XP-C cells were genetically corrected *ex vivo* (see text for details). Cells were then irradiated using 1500 J/m^2^ UVB (about two minimal erythemal doses in a control individual) and processed for autoradiography. High numbers (about 50–60) of black grains over nuclei are representative of the normal NER capacity. Note the quasi absence of grains in parental (XP-C) keratinocytes and their restoration at the level of WT cells in corrected keratinocytes (XP-C + *CD24-XPC*). (**B**) Skin regenerated using the same cells as in (**A**) were irradiated *in vivo* using 800 J/UVB m^2^ and processed for CPD immunolabeling immediately and 96h after irradiation. Note that the persistence of lesions in skin regenerated from parental XP-C keratinocytes (green label over nuclei), and the complete clearing of CPDs 96 h after irradiation in WT and in genetically corrected XP-C skin (XP-C + *CD24-XPC*). These observations show that CD24 selection of genetically corrected XP-C keratinocytes does not alter their fate *in vivo*. Bar, 150 μm.

To face the challenge of selection in XP epidermal cells, we used a natural cell surface molecule, CD24, expressed in post-mitotic, suprabasal keratinocytes. Onset of CD24 expression coincides with the loss of keratinocyte clonogenicity [76]. To the opposite, ectopic expression of CD24 at the surface of clonogenic keratinocytes allowed their immuno selection and rapid enrichment of holoclones during serial propagation [77]. Using this procedure we could reintroduce the XPC cDNA in XP-C keratinocytes and select transduced populations to homogeneity [78]. Full DNA repair and cell survival were recovered in transduced XP-C keratinocytes exposed to UVB radiations (Figure 2). The presence of stem cells in transduced populations was ascertained by clonal analyses over serial propagation of isolated holoclones. Encouragingly, DNA repair capacity and UV cell survival of corrected XP-C cells remained at WT levels for more than 100 population doublings. Transducing and selecting stem cells thus neither interfered with their growth capacity nor with the expression of their morphogenetic program. Indeed, they fully developed normal epidermis *in vitro* and *in vivo* after long term skin regeneration on the mouse. Further assessment of safety by ligation-mediated PCR, did not reveal retroviral (RV) insertions within coding sequences, or insertions closer than 10 kb of the transcription start. Furthermore, sequence integrations analyses in the host genome at either early or high passages failed to reveal amplification of specific sites of RV integration.

Altogether, our observations fulfill mandatory specifications inherent to corrective gene transfer in XP-C keratinocytes. As for any approach to corrective gene transfer, the immune response of the host against the therapeutic molecule is difficult to predict. The strictly nuclear location of XPC should limit its presentation to Langerhans cells, the dendritic cells of epidermis. However, the activation of patients’ lymphocytes in the presence of the therapeutic protein needs to be assessed before grafting of the genetically corrected epidermis. Together, our observations strongly stimulate research towards *ex vivo* cutaneous gene therapy of the XP disease and of other orphan genodermatoses such as epidermolysis bullosa.

## 11. Conclusions

Even though stem cells of the skin cannot be easily recognized due to the absence of specific markers, they can be cultured and genetically modified for therapeutic purposes. Our investigations in the XP show that the progeny of a single transduced epidermal stem cell may regenerate several square meters of cultured epithelia [5,78,79]. Further improvement of vectors, as well as prediction of immunogenic reactions, should pave the way towards reconstructive surgery using genetically keratinocytes. In the future, DNA microsurgery using modified meganucleases could also contribute to repair defective genes under their own regulatory sequences. Pending proper homologous recombination in stem cells, gene microsurgery will certainly represent the ideal procedure to improve care of XP-C patients. Some of the current specifications such as selection of transduced cells and the elimination of off-target recombination events will remain mandatory for technological applications to the bedside, and will continue to contribute to our basic knowledge.

## Figures and Tables

**Table 1:** Representative markers used for enrichment of stem cells of the interfollicular epidermis. High or low indicate levels of expression in stem keratinocytes relative to other basal keratinocytes; ESC, epidermal stem cells; EGF, epidermal growth factor; MCSP, melanoma-associated chondroitin sulfate proteoglycan; Lrig 1, leucine-rich repeats and immunoglobulin-like domains 1.

Marker	Function	Relative level in ESC	Reference
β1 integrin	Anchoring of basal keratinocytes	high (×2)	[31,37]
α6 integrin	Anchoring of basal keratinocytes	high	[32,33]
CD71	Transferrin receptor	low	
Delta1	Notch1 ligand	high (×2)	[38]
Desmogleine 3	Desmosomal component	Low (/4)	[39]
EGF-R	EGF receptor	low	[34]
MCSP	Proteoglycan	high (×7)	[40]
Lrig 1	EGF-R antagonist	high (×7)	[41,42]

**Table 2:** Main human genodermatoses. CL, cornified layers; GL, granular layer; SPL, spinous layer; BL, basal layer; BMZ, basement membrane zone; BM, basement membrane; Kx, Keratin X; LEKTI, Lympho-epithelial kazal type related inhibitor; CE/CL, cornified envelope/cornified layer; IF, interfollicular; HD, hemidesmosome; XPx, xeroderma pigmentosum X.

Disease	Gene	Protein	Protein function	Site of expression	Symtoms
Netherton syndrome	*SPINK 5*	LEKTI	Serine protease inhibitor	CL	Desquamation/barrier
Vulgaris ichtyosis	*FLG*	Filaggrin	CE/CL component	GL	Terminal differentiation/Barrier
Lamellar ichtyosis	*TGM1*	Transglutaminase	Cross linking enzyme	GL	Terminal differentiation/Barrier
X-linked ichtyosis	*STS*	Aryl-*C*-steroid/sulfatase	Steroid sulfatase	GL	Terminal differentiation/Barrier
Epidermolysis Hyperkeratotis	*KRT1/KRT10*	Keratins K1/K10	IF	SPL	Mechano bullous disease
Epidermolysis Bullosa Simplex	*KRT5/KRT14*	Keratins K5/K14	IF	BL	Mechano bullous disease
Jonctional Epidermolysis Bullosa	*ITGA6*, *ITGB4*	Integrins α6, Integrin β4	Membrane receptors of HD	BMZ	Mechano bullous disease
	*Col17A1*	Collagen 17/BP180	Anchoring filaments of HD	BMZ	Mechano bullous disease
	*LAMA3*, *LAMB3*, *LAMC2*	Laminin 332	BM components/Anchoring filaments of HD	BMZ	Mechano bullous disease
Dystrophic Epidermolysis Bullosae	*Col7A1*	Collagen 7 α1chain	Anchoring fibrils of HD	BMZ	Mechano bullous disease
Xeroderma pigmentosum	*XPA* to *XPG*	XPA to XPG	Repair of UV-induced DNA lesions	Ubiquitous	Photosensitivity/Skin cancer
Xeroderma pigmentosum variant	*POLH*	DNA polymerase η	Replicative translesion synthesis	Ubiquitous	Photosensitivity/Skin cancer
